# Visual Identity, Social Branding, and Advocacy: Development of the PEN‐Plus Logo for Severe Non‐Communicable Disease Care in Nepal

**DOI:** 10.1002/puh2.70242

**Published:** 2026-05-11

**Authors:** Dhurba Khatri, Shishir Paudel, Yamuna Chhetri, Bhakta Bahadur K. C., Rudra Prakash Hada, Sheela Shrestha, Leela Khanal, Sandeepa Karki

**Affiliations:** ^1^ Kathmandu Institute of Child Health (KIOCH) Kathmandu Nepal; ^2^ National Centre for Epidemiology and Population Health The Australian National University Canberra Australia; ^3^ Ministry of Health and Population Kathmandu Nepal; ^4^ National Health Education, Information and Communication Center Kathmandu Nepal; ^5^ Independent Researcher Kathmandu Nepal

## Abstract

Non‐communicable diseases (NCDs), such as type 1 diabetes, rheumatic heart disease, and sickle cell disease, impose significant health and social burdens in Nepal, particularly among children and young adults. To strengthen visibility, reduce NCD‐related stigma, and foster engagement with the PEN‐Plus program, a participatory design process was adopted to develop a national logo. This article outlines and critically reflects on the rationale, participatory development process, and symbolism behind the PEN‐Plus Nepal logo, situating it within the broader field of health communication and social branding. Drawing on behavioral science theories and participatory design principles, the PEN‐Plus logo was co‐created with patients, health workers, government representatives, and communication experts. Ministry of Health and Population, National Health Education, Information and Communication Center officially endorsed the PEN‐Plus logo, which now serves as a unifying symbol to raise awareness, foster community ownership, and support long‐term advocacy for equitable severe NCD care in Nepal.

## Graphical abstract

Nepal's PEN‐Plus logo, developed through a participatory process, serves as a unifying visual identity for severe non‐communicable disease care. Integrating social branding and behavioral insights, it uses symbolic elements to raise awarness to reduce stigma, promote inclusion, and address barriers to care. It fosters community engagement, builds trust, and aligns stakeholders toward equitable, accessible, and sustainable health services for children and young adults living with severe NCDs.

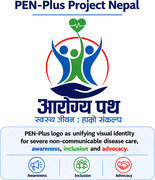

**Abbreviations**
DoHS: Department of Health Services
FCHV: Female community health volunteer
IEC: Information, education, and communication
KIOCH: Kathmandu Institute of Child Health
MoHP: Ministry of Health and Population
NCDI: non‐communicable diseases and injuries
NHEICC: National Health Education, Information and Communication Center
NHTC: National Health Training Center
PEN: Package of Essential Non‐Communicable Disease Interventions
PEN‐Plus: Package of Essential Non‐Communicable Disease Interventions‐Plus
SCD: sickle cell disease
SCT: social cognitive theory
TPB: theory of planned behavior
UNICEF: United Nations International Children's Emergency Fund
WHO: World Health Organization

## 1 Background

Non‐communicable diseases (NCDs) have emerged as a critical public health challenge worldwide, and Nepal is no exception. NCDs account for over 70% of all deaths in the country, with profound economic and social consequences [1]. Although NCDs are frequently perceived as illnesses linked to adult behaviors such as smoking, alcohol use, or physical inactivity, this preconceived notion overlooks a critical reality, where many severe forms of NCDs begin in childhood or adolescence and are not preventable through lifestyle changes alone [2, 3]. Conditions such as type 1 diabetes mellitus, sickle cell disease (SCD), thalassemia, rheumatic heart disease, and childhood cancers impose a lifelong burden that often leads to early mortality, social stigma, and financial hardship, especially in low‐resource settings like Nepal [3, 4, 5].

Recognizing this gap, the Ministry of Health and Population (MoHP) of Nepal, in collaboration with the Kathmandu Institute of Child Health (KIOCH), and with financial support from the NCDs and Injuries (NCDI) Poverty Network secretariat at the Center for Integration Science, Brigham and Women's Hospital, introduced the PEN‐Plus program in 2021 [6]. Building upon the foundation of the World Health Organization's Package of Essential NCD Interventions (WHO‐PEN), which primarily targets adult‐onset NCDs such as hypertension and type 2 diabetes [7, 8], the PEN‐Plus initiative extends care to include severe and chronic conditions affecting younger populations [4, 9]. By decentralizing specialized services to district‐level hospitals, the program seeks to reduce the over‐reliance on overcrowded tertiary centers and improve access for underserved communities [6, 10, 11].

Despite the promise of PEN‐Plus, public awareness and engagement around severe NCDs remain limited and could hinder uptake and continuity of care. Social stigma, misinformation, and a lack of recognition of the lifelong needs of individuals living with these conditions further contribute to the invisibility of the issue in public discourse. In this context, visual identity and branding can serve as powerful tools for public health communication. Especially for programs that aim to shift narratives, mobilize support, and increase community engagement, symbols like logos can strengthen visibility and legitimacy. Consistent with practices in global health communication, the PEN‐Plus Nepal team developed a unique logo as both a symbol of commitment and a medium for social advocacy.

### 1.1 Rationale for Visual Identity and Social Branding of PEN‐Plus

In public health communication, branding is a strategic tool to build meaningful relationships between communities and health programs, services, or messages. It involves the intentional use of names, signs, colors, and symbols to communicate value, enhance recognition, and encourage engagement [12, 13]. Importantly, the symbolic elements of branding are not merely decorative; they differentiate health initiatives, enhance recall, and create emotional resonance [14]. Social branding is an innovative approach in health promotion that draws from both behavioral science theories and marketing theories to influence how people think, feel, and act concerning health and health programs. It also leverages insights from psychology and sociology to guide behavior change while applying strategic principles from commercial and social marketing to package and deliver messages in emotionally resonant and culturally appropriate ways.

Behavioral theories such as social cognitive theory (SCT) and the theory of planned behavior (TPB) further reinforce the importance of branding [15, 16]. The SCT developed by Bandura emphasizes observational learning, modeling, and self‐efficacy in influencing behavior [15]. In social branding, SCT suggests that visuals and symbols can model aspirational behaviors and strengthen individuals’ belief in their ability to adopt them. For instance, when a logo represents empowered individuals managing chronic illness, it illustrates desired behavior and conveys that such behavior is achievable. The TPB, as articulated by Ajzen [16] and extended by Fishbein and Yzer [17], highlights how intention, shaped by attitudes, perceived norms, and perceived control, guides behavior. Thus, a strong, resonant brand can positively shape attitudes, signal normative support, and foster a sense of control, thus supporting healthier decision‐making [18, 19]. Importantly, social marketing theory complements these behavioral theories by focusing on how principles of commercial marketing can be adapted for the social good and how product, price, place, and promotion could influence voluntary behavior change. In this model, branding is critical to the “product” and “promotion” elements: It shapes how people perceive the health service or behavior being offered, and how compellingly that offering is communicated. By integrating these theories, social branding helps create a cohesive narrative and emotional engagement, transforming abstract health goals into something concrete, visible, and familiar.

Globally, social branding has been effective in promoting healthier behaviors such as increased condom use, reduced tobacco and substance consumption, and other preventive practices [12, 18, 19, 20]. By aligning health messages with self‐image and cultural values, social branding enhances motivation and community ownership. When adapted to local contexts, it helps mobilize support and collective action [20]. In Nepal, branding has been pivotal in reinforcing public health messages, with the use of symbols and logos serving as powerful tools in several health initiatives. One prominent example is the logo of the female community health volunteer (FCHV) program, which features four circles in a sky‐blue background. Over time, this symbol has come to represent trust and commitment at the grassroots level, holding deep meaning among the general population as a trusted emblem of community‐based health services delivered by FCHVs [21]. Another notable example is the iodized salt campaign, where a logo depicting two children played a key role in reinforcing public awareness about iodine deficiency and its impact on child development. This simple visual device helped embed the importance of iodized salt into everyday behavior and public understanding [22]. These national examples demonstrate how strategically designed visual identities can embed public health messages into cultural practice and sustain behavior change. Drawing on this evidence, the PEN‐Plus Nepal recognized the need for a distinctive logo to strengthen advocacy for people living with severe NCDs. The logo was conceptualized not as a mere graphic, but as a strategic identity marker to reduce stigma, enhance visibility, and unify diverse stakeholders under a shared mission. The development process was designed to be participatory, inclusive, and context‐sensitive, engaging patients, health professionals, government stakeholders, and communication experts.

### 1.2 Process of Logo Development

**FIGURE 1:**
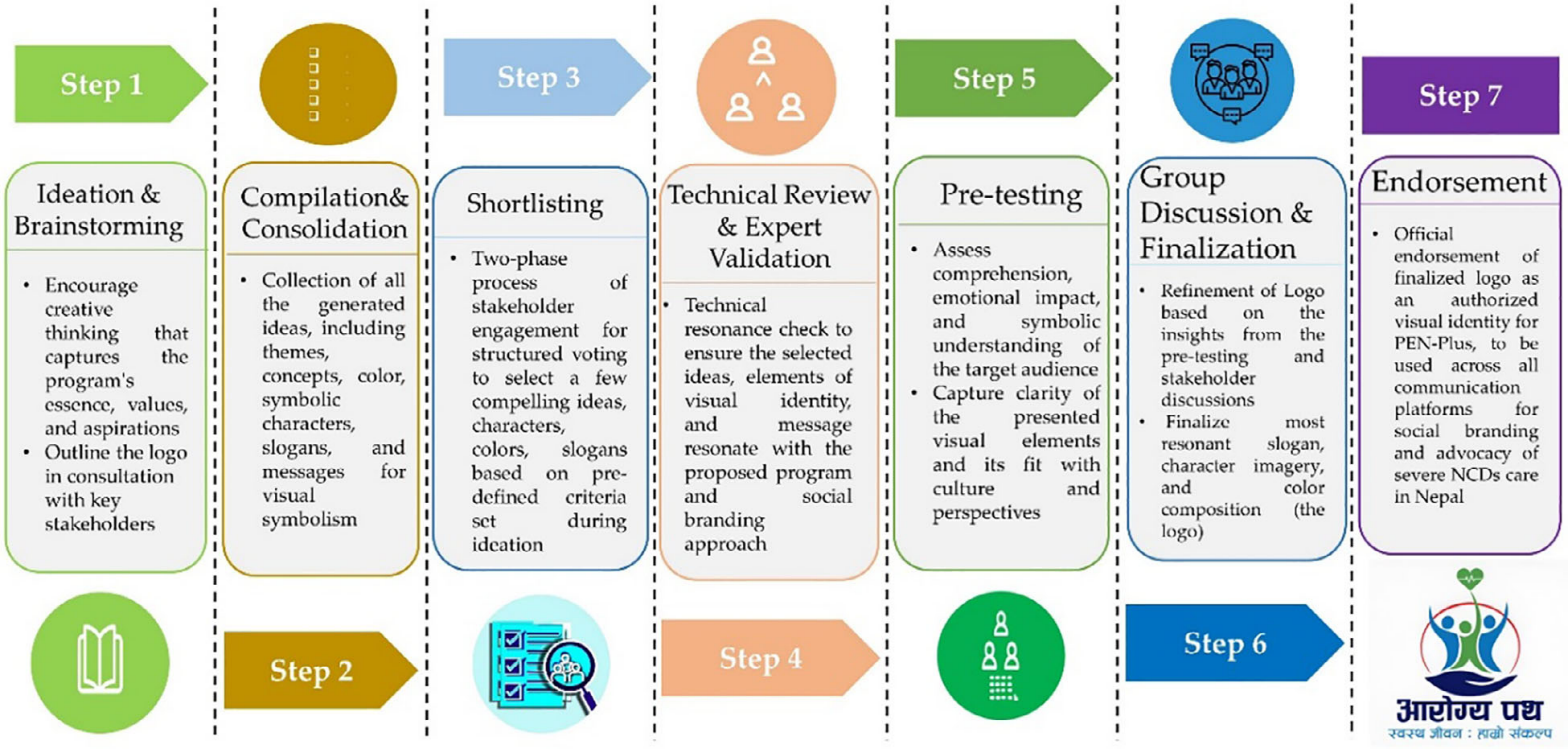
Process of PEN‐Plus Nepal logo development.

The logo was developed using a participatory design approach grounded in social branding theory and principles of community‐engaged health communication. The development of the PEN‐Plus Nepal logo was undertaken as a collaborative and sequential process, designed to reflect the program's values, mission, and aspirations. It incorporated perspectives from a wide range of stakeholders, including service providers, program implementers, adolescents with and without NCDs, government representatives, and health professionals, ensuring that the final product was both contextually grounded and socially resonant. The logo development process was strategically planned and coordinated by the PEN‐Plus core team, which comprised a multidisciplinary group of healthcare professionals with expertise in NCDs, academia, and health promotion and education (Figure 1).

#### 1.2.1 Step 1: Ideation and Brainstorming

The initial phase focused on fostering creative thinking around the essence of the PEN‐Plus program, considering its goals, values, and the communities it serves. This initial phase gathered diverse perspectives on possible names, slogans, symbols, and characters that could capture the essence of PEN‐Plus. Input was solicited from a wide pool of stakeholders at multiple levels. At the community level, adolescents with and without NCDs were consulted. At the facility level, PEN‐Plus clinic staff and healthcare providers from district hospitals contributed ideas. At the provincial and national levels, contributions were sought from technical experts, program managers, and institutional stakeholders. These included officials from the MoHP, the National Health Education, Information and Communication Center (NHEICC), United Nations International Children's Emergency Fund (UNICEF), logo design professionals, and public health communication experts. Additional contributions were provided by seven trained public health professionals, five national NCD consultants, three clinicians, two community medicine specialists, and a health journalist. Adolescents and patients living with severe NCDs contributed perspectives on emotional resonance and stigma; health workers and clinicians provided input on clinical appropriateness; government representatives ensured policy alignment; and communication experts guided visual and messaging clarity. Feedback was collected through emails, structured consultations, and group discussions.

#### 1.2.2 Step 2: Compilation and Consolidation

All proposed ideas, including names, taglines, themes, symbolic characters, and messages, were compiled into a comprehensive list (Supporting Information section) that contains the raw suggestions generated during the participatory consultations and subsequent shortlisting process. The design team then developed a structured evaluation matrix, guided by pre‐defined criteria: clarity, cultural and linguistic relevance, emotional appeal, alignment with PEN‐Plus principles, and recognizability. This process was consultative and iterative rather than formal qualitative analytic exercise. This ensured only contextually meaningful and technically appropriate elements moved forward.

#### 1.2.3 Step 3: Shortlisting Through Stakeholder Engagement

A two‐phase shortlisting process was carried out. First, provincial and local stakeholders in the Lahan district participated in a structured voting and review session. This included district health officers, municipal‐level representatives, health facility staff, and community‐based actors. In the second round, a similar participatory session was held in Dhulikhel with national‐level stakeholders, including NHEICC, the National Health Training Center (NHTC), UNICEF Nepal, KIOCH, and NCD consultants. Structured voting based on agreed criteria yielded six shortlisted components for refinement.

#### 1.2.4 Step 4: Technical Review and Expert Validation

The PEN‐Plus Technical Working Group, consisting of senior public health professionals, communication specialists, and government program leads, reviewed the shortlisted concepts. These technical resonance checks evaluated alignment with national strategies, coherence with PEN‐Plus goals, and feasibility for use in health promotion and mass communication.

#### 1.2.5 Step 5: Pre‐Testing With Target Audiences

Draft logos were tested with PEN‐Plus health workers, service providers, health administrators, and people living with severe NCDs. Participants were engaged through short cognitive interviews in which they were asked to interpret the logo's colors, symbols, name, and tagline. This qualitative process aimed to capture emotional responses, clarity of understanding, and perceived cultural fit.

#### 1.2.6 Step 6: Group Discussion and Finalization

The final design refinement was conducted on the basis of the insights generated from the pre‐testing phase and further stakeholder deliberations. The design team and selected stakeholders revisited options to confirm the most resonant slogan, imagery, and color composition. Special care was taken to ensure the logo was emotionally impactful, visually distinct, culturally appropriate, and adaptable across different communication platforms.

#### 1.2.7 Step 7: Official Endorsement

The finalized logo was formally endorsed by the NHEICC, following administrative review and approval processes, establishing it as the official visual identity of the PEN‐Plus program in Nepal. This endorsement represents a programmatic outcome of the development process. With this endorsement, the logo now serves as the authorized visual identity for PEN‐Plus and will be used across all communication platforms, including media campaigns, printed IEC (Information, Education, and Communication) materials, digital health messages, national reports, and awareness‐raising events. This formal recognition not only validates the participatory design process but also ensures that future messaging under the PEN‐Plus brand will carry a unified and recognizable symbol reflective of the program's values.

### 1.3 Decoding the Logo: Symbolism and Meaning

**FIGURE 2:**
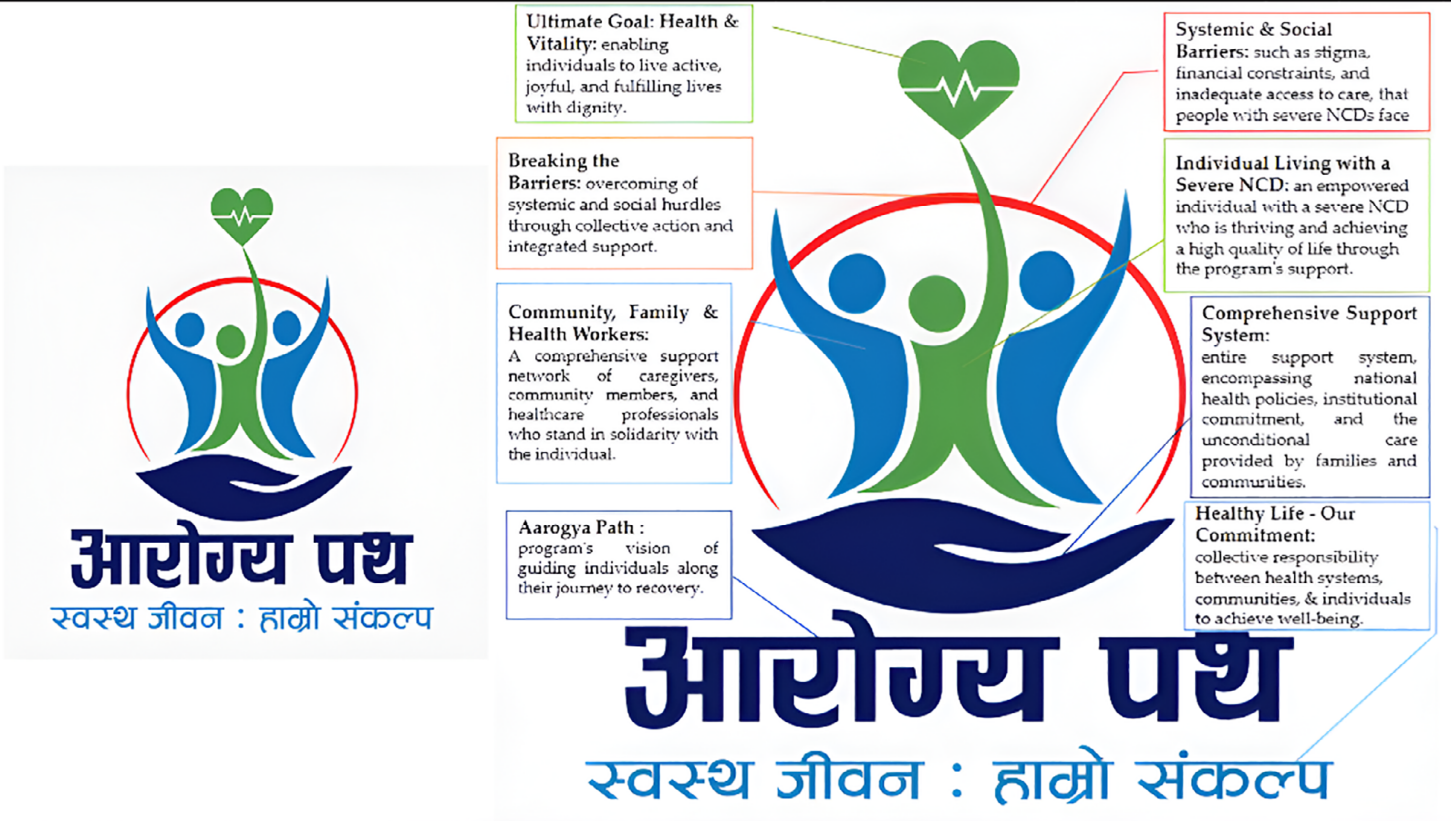
PEN‐Plus Nepal logo symbolism and meaning.

The PEN‐Plus Nepal logo was designed as a powerful visual metaphor that encapsulates the values, mission, and aspirations of the program. Every element in the logo was deliberately selected to communicate a multi‐layered message around care, equity, and collective action for individuals living with severe NCDs. It serves not only as an identity marker but also as a visual tool for advocacy, awareness, and community mobilization (Figure 2).

The test at the base of the logo “Aarogya Path” (आरोग्य पथ) roughly translates to “Path to Health” or “The Road to Recovery,” reflecting the vision of guiding individuals through their health journey with compassion and resilience. “Aarogya Path” reinforces the concept that health is a journey where one must be supported by a conducive environment and responsive systems. Beneath this, the slogan “Swastha Jeeban: Hamro Sankalpa” (स्वस्थ जीवन: हाम्रो संकल्प), meaning “Healthy Life: Our Commitment,” emphasizes both individual and collective responsibility of community and organization in achieving and maintaining well‐being. It reinforces the notion that health is not only a service but a shared social contract, where every stakeholder has a role to play.

The slogan is intentionally open to interpretation based on the audience's professional and social standpoint. For healthcare providers, it represents a pledge to deliver respectful, patient‐centered, and quality care. For policymakers, it implies accountability in designing and implementing inclusive health policies that integrate health in all sectors. For communities and individuals, it signifies the importance of adopting healthy behaviors, adhering to prescribed treatment regimens, and actively participating in one's care. Thus, commitment must be upheld by both health systems and communities or individuals.

The outstretched hand at the bottom of the logo represents the comprehensive care and support system upon which the PEN‐Plus program is built. It is a system‐level support that includes national health policies, public health infrastructure, trained healthcare professionals, community‐based support networks, and the unconditional care provided by families. The hand signifies protection, nurturing, and institutional commitment, anchoring the individual within a supportive environment that prioritizes access, equity, and continuity of care.

Above the hand, three human figures emerge in a unified pose, symbolizing togetherness, inclusion, and shared action. The blue figures on either side represent the community, families, and health workers (collectively caretakers), who are the broader network of people, who provide emotional, informational, and practical support to the people living with severe NCDs. At the center stands a green figure, representing an individual living with a severe NCD who has achieved improved quality of life with appropriate care. The symmetrical and uplifting posture of these three figures embodies encouragement, acceptance, and shared responsibility. Their raised arms symbolize empowerment and triumph, showing that individuals can thrive when surrounded by compassion, care, and understanding. Importantly, their convergence conveys a message that people living with NCDs are not alone and the condition is not contagious. Building trust, promoting inclusion, and ensuring care must be collaborative efforts between individuals, communities, and the health system.

The red semi‐circular arc that encircles the figures signifies the barriers faced by individuals with severe NCDs and their caregivers. These barriers can include physical, social, or systemic hurdles, such as myths and misconceptions, delayed health‐seeking behavior, lack of information, inadequate access to quality care, financial constraints, and societal stigma. These challenges often limit access to timely and appropriate care. Notably, the red arc is interrupted at the point where the green figure ascends, symbolizing the breaking of these barriers through strong support systems, informed care, and collective resolve. This intersection is a key visual message symbolizing that the barriers can be overcome through collective effort and integrated support.

Crowning the logo is a heart‐shaped icon with an electrocardiogram rhythm, symbolizing life, vitality, and good health. This icon represents the goal of the PEN‐Plus program, which is to enable individuals with severe NCDs to live active, joyful, and fulfilling lives while acknowledging and managing their conditions. The presence of the heart and the cardiac rhythm reinforce the importance of continued monitoring, quality care, and living with dignity despite chronic conditions. It is a message of hope, possibility, transformation, and the value of every heartbeat.

The logo's design not only visually encapsulates the program's philosophy but also communicates a larger message of hope, partnership, and empowerment. The point of intersection, where the green figure pierces the red arc (barriers), marks a pivotal moment of overcoming systemic barriers, enabled by compassionate support and community engagement. This represents the journey from isolation and stigma to inclusion and dignity. This symbolic breakthrough is a visual call to action, reminding all stakeholders that equity in health is achievable when care is inclusive, accessible, and rooted in solidarity. It functions as a powerful visual advocacy tool that challenges stigma, inspires action, and unites people around the shared mission of improving lives through inclusive NCD care. As implementation expands, future monitoring of awareness, recognition, and stakeholder engagement may further inform its long‐term effectiveness within social and behavior change communication (SBCC) strategies.

### 1.4 Logo as a Tool for Advocacy and Action

The PEN‐Plus Nepal logo serves as a strategic communication tool to raise awareness, promote inclusion, and catalyze collective action around severe NCDs. In Nepal, where severe NCDs often remain under‐recognized and under‐resourced, the logo strengthens visibility and legitimacy for this neglected area of public health. Communicating complex ideas in a clear, emotionally resonant, and culturally grounded approach enables diverse audiences to engage with the program's mission. Representing individuals with severe NCDs alongside families, communities, and care systems, the logo reinforces values of empathy, shared responsibility, and hope. By humanizing those affected and portraying them as empowered individuals supported by collective care, it challenges stigma and misconceptions. When applied consistently across health facilities, campaigns, training, and SBCC materials, the logo builds familiarity, trust, and recognition, which are the elements that are essential for long‐term engagement with health services.

The logo development process also revealed several challenges and lessons relevant for future initiatives. Balancing emotional resonance with clinical accuracy requires careful negotiation between patient perspectives and technical health messaging. Managing diverse stakeholder expectations, including those of patients, clinicians, government representatives, and communication experts, necessitated iterative dialogue and compromise. Representing multiple severe NCD conditions within a single visual identity posed difficulties in avoiding disease‐specific symbolism while maintaining inclusivity. These experiences highlight the importance of participatory design, flexibility, and ongoing refinement when developing visual identities for complex health programs.

As the Government of Nepal expands NCD awareness activities, such as recognizing mid‐February to mid‐March as NCD Awareness Month and conducting screening campaigns at the local level, there remains no universal symbol for the national NCD program. In this gap, the PEN‐Plus logo provides a much‐needed unifying identity for advocacy, policy, and service delivery. Over time, it can evolve into a social norm symbol, reframing essential services like insulin access for type 1 diabetes or blood transfusions for SCD not as privileges but as basic health rights. By associating the logo with equitable access and social justice, particularly for underserved populations disproportionately affected by certain severe NCDs like SCD, it can reinforce public expectations and policy commitments. Thus, the logo has potential to become a normative cue for dignity, inclusion, and accountability in NCD care.

Beyond Nepal, the PEN‐Plus logo holds broader global relevance. Health communication in many low‐ and middle‐income countries faces barriers related to language, literacy, cultural interpretation, and resource constraints [23, 24]. A well‐designed visual identity, informed by participatory methods and grounded in behavioral theory, can transcend these barriers and provide a unifying symbol. Such symbols create community ownership and motivate stakeholders to align around common goals. Globally, visual branding in health has been instrumental in generating momentum for initiatives such as HIV/AIDS awareness, maternal and child health, and immunization campaigns [12, 18, 19, 20]. The PEN‐Plus logo illustrates how similar approaches can be adapted for severe NCDs, especially among children and adolescents, within low‐ and middle‐income country contexts where health communication must balance cultural relevance, resource constraints, and health system realities. It offers a replicable model for culturally rooted, community‐driven communication strategies that elevate underrepresented health priorities. In essence, the logo is not only a program emblem but also a dynamic tool for advocacy, behavior change, and systems strengthening, bridging technical programs with public perception and enabling more inclusive and sustainable engagement.

As a perspective account of a participatory branding initiative, this article does not present formal empirical evaluation of the logo's impact on awareness or behavioral outcomes. A key limitation of the logo is that symbolic interpretation may vary across cultural, linguistic, and age groups, and a single visual identity cannot fully capture the diversity of experiences across different severe NCD conditions or overcome structural barriers to care. The logo should therefore be understood as one component within a broader health communication and systems‐strengthening strategy.

## 2 Conclusion

The PEN‐Plus Nepal logo serves as a powerful communication tool that promotes visibility, reduces stigma, and fosters collective commitment to severe NCD care. Its development through a participatory and context‐sensitive process illustrates how visual identity can support public health advocacy, both locally and globally. Beyond its local impact, it provides a model for other countries seeking innovative, low‐cost communication strategies to legitimize neglected health conditions and mobilize collective action. It is recommended that future NCD programs in LMICs could integrate participatory visual branding into their social and behavior change communication strategies to strengthen visibility, reduce stigma, and support sustained policy advocacy.

## Author Contributions

**Dhurba Khatri**: conceptualization, visualization, validation, project administration, methodology, writing – review and editing. **Shishir Paudel**: conceptualization, visualization, project administration, methodology, writing – original draft, writing – review and editing. **Yamuna Chhetri**: project administration, visualization, writing – review and editing. **Bhakta Bahadur K. C**.: visualization, validation, writing – review and editing. **Rudra Prakash Hada**: visualization, writing – review and editing. **Sheela Shrestha**: visualization, validation, writing – review and editing. **Leela Khanal**: visualization, writing – review and editing. **Sandeepa Karki**: conceptualization, visualization, validation, methodology, project administration, supervision, writing – review and editing.

## Funding

Funds for the consultation and technical session for development of the logo were provided by UNICEF Nepal.

## Ethics Statement

The authors have nothing to report.

## Consent

The authors have nothing to report.

## Conflicts of Interest

The authors declare no conflicts of interest.

## Supporting information




**Supplementary File1**: puh270242‐sup‐0001‐SupMat.docx

## Data Availability

Data sharing is not applicable to this article as no datasets were generated or analyzed during the current study.
