# Supplementary material for: Visual Identity, Social Branding, and Advocacy: Development of the PEN‐Plus Logo for Severe Non‐Communicable Disease Care in Nepal
Source: Public Health Chall. 2026 May 11;5(2):e70242. doi: 10.1002/puh2.70242 (PMC13159710; doi:10.1002/puh2.70242)

**Program Name**

**Characters of the Program Name**

The program name should be appealing, non-technical, easily understandable, and catchy, while effectively conveying the program's impact. It should resonate with the target audience and align with the program's objectives

The ideal name should:

- Reflect the impact and significance of our program in addressing non-communicable diseases (NCDs) and neurodevelopmental disorders among children and adolescents.
- Be non-technical and easily comprehensible by people of all backgrounds and ages.
- Carry a sense of hope, empowerment, and community engagement

**Collection of the Suggested Names:**

| **SN** | **प्रस्ताबित - नाम** (Purposed Names for logo) | **Rough Translation** | **SN** | **प्रस्ताबित - नाम** (Purposed Names for logo) | **Rough Translation** | **SN** | **प्रस्ताबित - नाम** (Purposed Names for logo) | **Rough Translation** |
| --- | --- | --- | --- | --- | --- | --- | --- | --- |
|  | आशाको किरण | Ray of Hope |  | सङ्कल्प | Commitment |  | आरोग्य पथ | Path to Health/ wellbeing |
|  | आशाको केन्द्र | Center of Hope |  | आरोग्य निर्माण | Health Development |  | आरोग्य मार्ग | Road to Health |
|  | नवजिवन | New Life |  | आरोग्य सक्षम | Health Empowered |  | नयाँ ज्योति | New Light |
|  | NCD कवच | NCD Shield |  | उमिद | Hope |  | सुस्वास्थ्य अभियान | Good Health Campaign |
|  | NCD प्रतिरोध | NCD Resistance |  | सम्बभ | Possible |  |  |  |
|  | NCD निवारण | NCD Elimination |  | बल कल्याण | Strength & Welfare |  | दिर्घ सेवा | Service for Chronic Conditions |
|  | संगै हिड्यौ | Walk Together |  | स्वास्थ्य सेतु | Health Bridge |  | पुर्नजीवन | Rebirth / Revival |
|  | जित्न सकिन्छ | It can be won |  | नयाँ दिशा | \| New Direction \| \| --- \| |  | दिगो सेवा | Sustainable Chronic Service |
|  | सम्बभ छ | It is possible |  | आरोग्य मित्र | Healthy Friend |  | जीवन बरदान | Life's Blessing |
|  | हासेर बाचौ | Let’s live with Laughter |  | जीवन रक्षा | Protecting life |  | मृत्युञ्जय | Conquering Death |
|  | म सक्छु | I Can |  | जीवन दर्पण | Mirror of Life |  | संजीवनी | Sanjeevani (Life-giving elixir) |
|  | शतावारी | Shatavari (Herbal name, often used symbolically for health) |  | आरोग्य कवच | Health shield |  | स्वास्थ्य साथी | \| Health Companion \| \| --- \| |
|  | शतपथ | Path of Century |  | जीवन सारथी | Life Companion |  | समर्थ संकल्प | Capable Commitment |
|  | मेरो विश्वास | My Belief |  | नयाँ प्रयास | New Attempt |  | निरमय नवयुग | Disease-Free New Era |
|  | प्रगतिपथ | Path of Progress/ Treatment |  | सम्भावना | Possibility |  | सामिप्यता | \| Closeness \| \| \| --- \| --- \| \|  \| |
|  | सुयोग्य | Deserving / Suitable |  |  |  |  |  |  |

Shortlisted Names

| **क्र.स.** | **प्रस्ताबित - नाम** (Purposed Names for logo) | **Rough Translation** |
| --- | --- | --- |
|  | आशाको किरण | Ray of Hope |
|  | सम्बभ छ | It Is Possible |
|  | हासेर बाचौ | Let’s Live with Laughter |
|  | दिगो सेवा | \| Sustainable Service \| \| --- \| |
|  | जित्न सकिन्छ | It Can Be Won |
|  | सामिप्यता | Closeness |
|  | स्वास्थ्य साथी | Health Companion |
|  | आरोग्य पथ | Path to Health |
|  | आरोग्य मित्र | Healthy Friend |
|  | नयाँ प्रयास | New Attempt |
|  | आरोग्य निर्माण | Health Development |

**List of Possible Tagline**

- स्वास्थ्य तन, सिर्जनशील मन - Healthy Body, Creative Mind
- जटिल छ तर सम्भव छ -It's Complex but Possible -
- म जित्छु मेरो रोग I Will Defeat My Illness
- स्वास्थ शरीर, सक्षम भविष्य Healthy Body, Empowered Future
- मेरो स्वास्थ्य, मेरो विश्वास My Health, My Belief
- मेरो स्वास्थ्य, मेरो अधिकार My Health, My Right
- स्वस्थ नेपाली: समृद्ध नेपाली Healthy Nepali: Prosperous Nepali
- जीवनको ज्योति: स्वास्थ्य प्रगति Light of Life: Progress Through Health
- स्वस्थ जीवन : हाम्रो संकल्प Healthy Life:L our commitment
- हार होइन लड्न सिकौँ Let’s Not Give Up, Let’s Learn to Fight
- स्वास्थ्यसेवामा समता Equity in Health Services
- सृष्टि होइन, दृष्टि बदलौ Don’t Change the World, Change the Way We See It
- हामी साथ छौ We Are With You, We are together
- My smile doesn't fade with my NCD
- I conquer NCD
- My problem is my strength, not my weakness.
- My conditions are my strength
- I'm the leader of my life and future.
- My Health, My Right
- Transforming Lives Beyond NCDs
- Prosperity Through Wellness
- Building Minds, Preventing NCDs
- Empowering Wellness, Preserving Tomorrow
- Nurturing Health, Sustaining Lives
- Sustainable Choices, Lasting Health
- Preserving Health, Protecting People
- Wellness in Harmony with Nature
- Proper Health Care for Generations
- Sustainable Solutions, Lifelong Well-being
- Healthcare for Today, Sustainability for Tomorrow
- Sustainable Paths to Wellness
- Beyond Quick Fixes, Toward Lasting Health
- Managing Health, Not Just Treating Illness
- The Road to Better Health
- Invest in Your Future Health Today
- Together, let's strengthen NCD Care
- Invest in lifelong care
- Prevention is better than cure
- Together we can end NCD BeatNCD
- Act on NCDs #ActOnNCD
- Equity in healthcare
- Meaningful engagement of people with lived experience
- Train and retain health workforce

Short Listed Tagline

- सृष्टि होइन, दृष्टि बदलौ - Don’t Change the World, Change Your Perspective
- जीवन गतिशील छ -Life Is Dynamic
- हामी साथ छौ - We Are With You
- स्वास्थ शरीर, सक्षम भविष्य Healthy Body, Empowered Future
- म जित्छु मेरो रोग I Will Defeat My Illness
- हार्न होइन लड्न सिकौं Don’t Learn to Lose, Learn to Fight
- मेरो सङ्कल्प : म सक्छु र जित्छु My Determination: I Can and I Will Win
- My Health, My Right
- Transforming Lives Beyond NCDs
- The Road to Better Health
- Invest in Your Future Health Today

**Draft Logo for Expert review and Discussion:**

**Color theme NCDIPC, WHO, and PEN-Plus reflection**


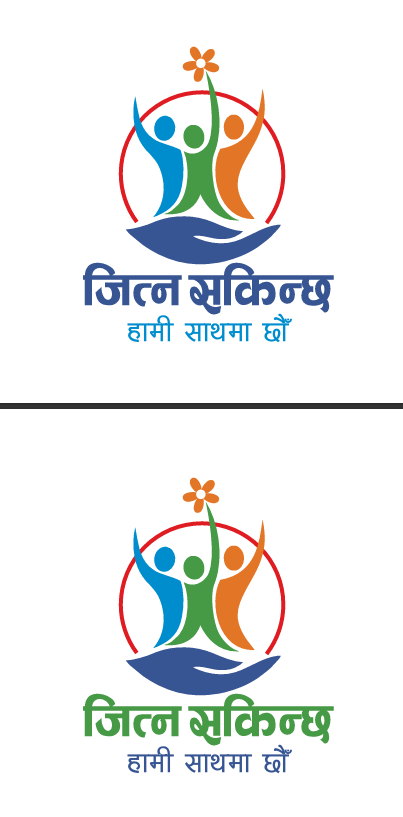

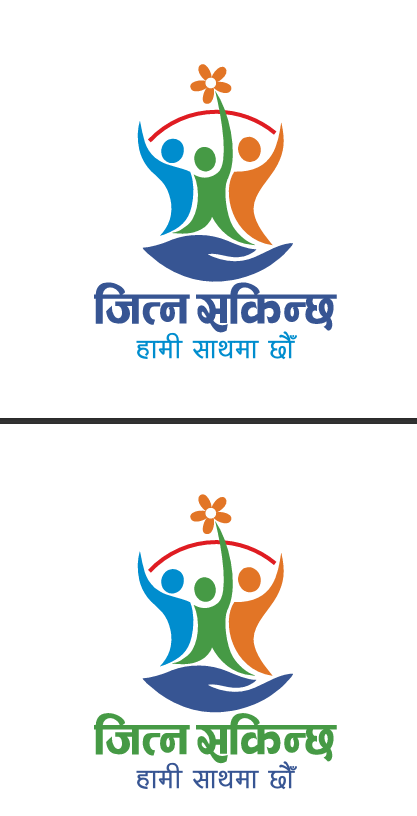

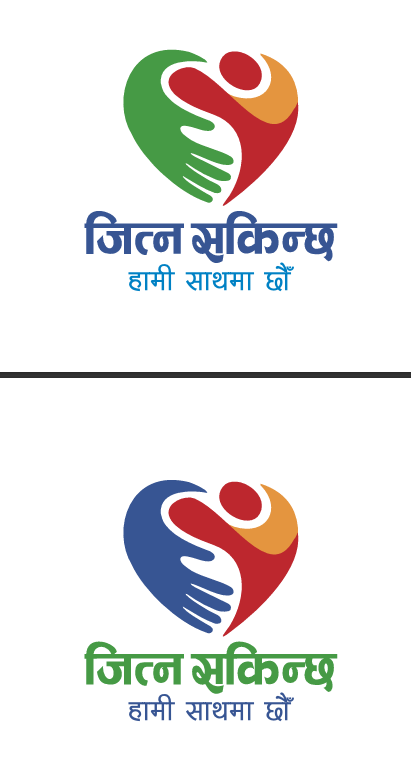

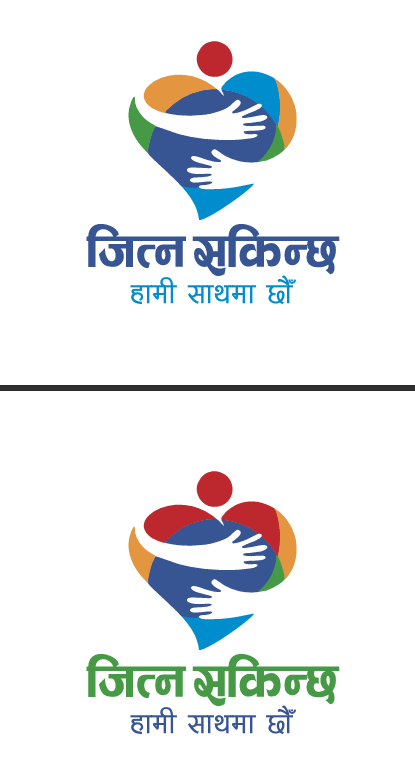

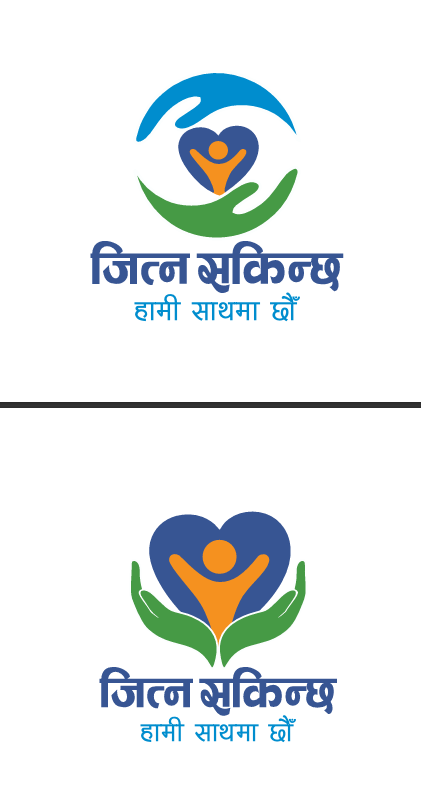


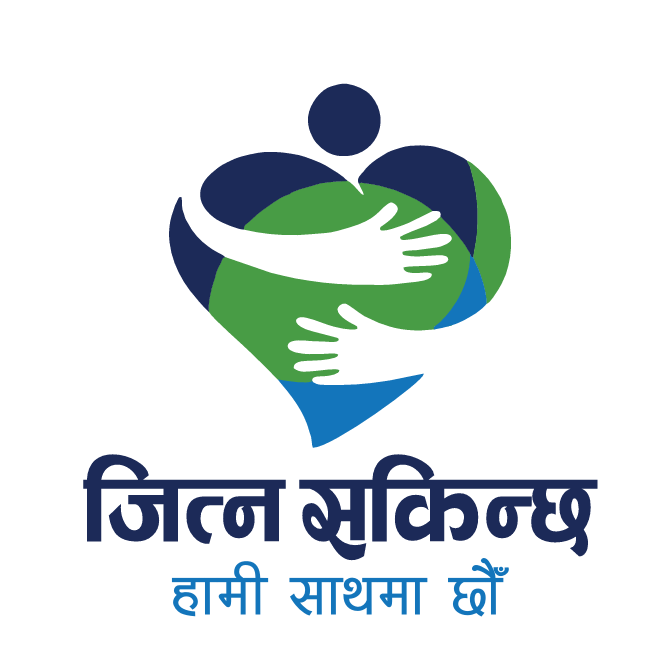

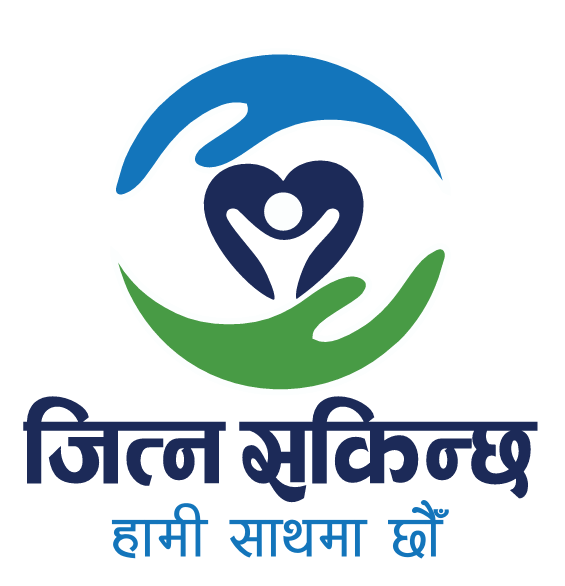

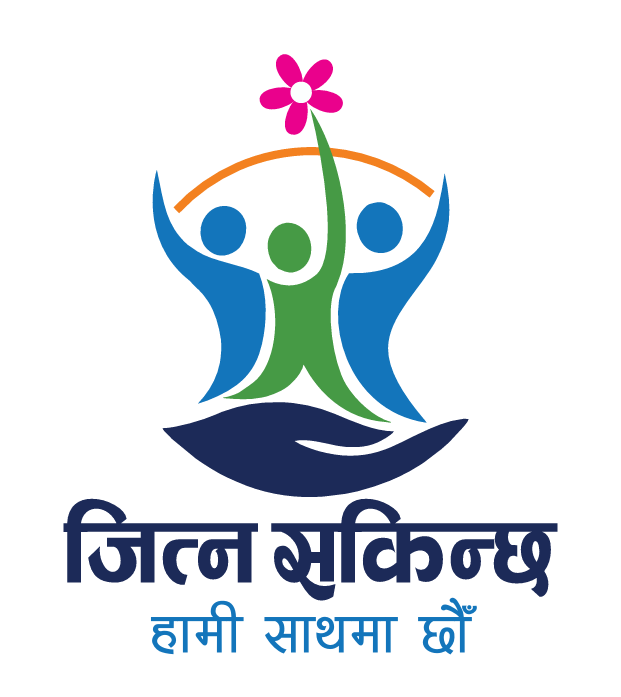

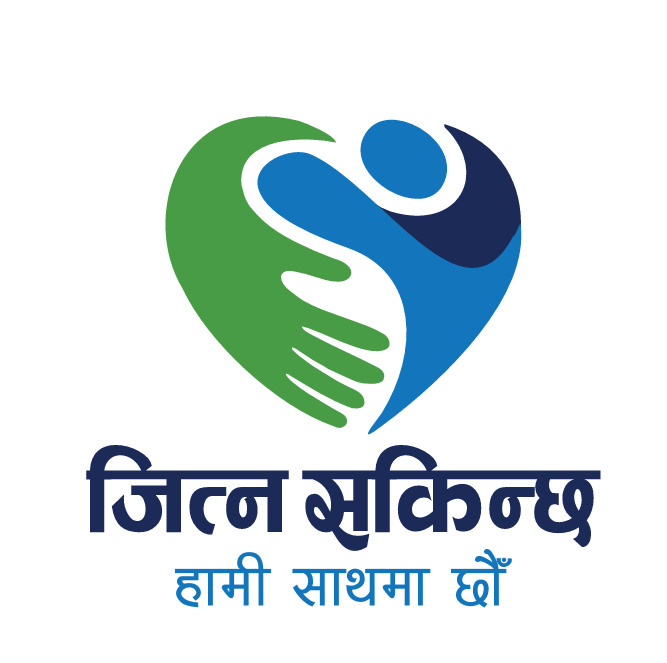


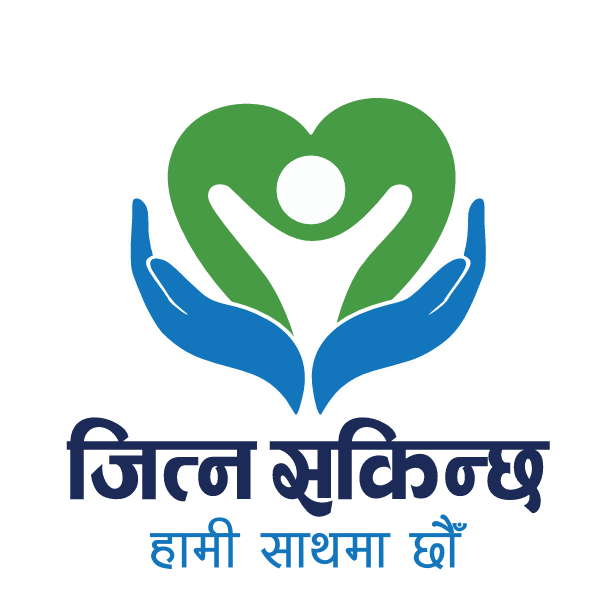

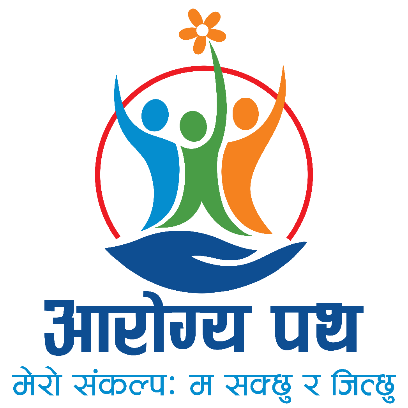

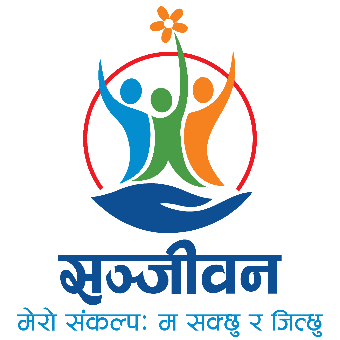


**Finalized Logo**


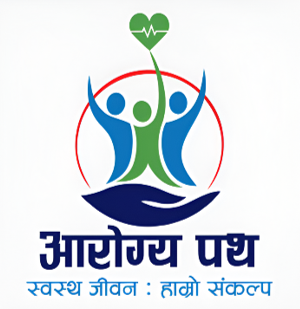

Supplement: Supplementary file 1 — Supplementary File1: puh270242‐sup‐0001‐SupMat.docx [file PUH2-5-e70242-s001.docx]
